# Seroprevalence of Hepatitis E Virus Infection among Blood Donors in Bulgaria

**DOI:** 10.3390/v13030492

**Published:** 2021-03-16

**Authors:** Magdalena Baymakova, Krasimira Terzieva, Rumen Popov, Elisaveta Grancharova, Todor Kundurzhiev, Roman Pepovich, Ilia Tsachev

**Affiliations:** 1 Department of Infectious Diseases, Military Medical Academy, 1606 Sofia, Bulgaria; 2 Center of Transfusion Hematology, Military Medical Academy, 1606 Sofia, Bulgaria; krasimirater@abv.bg (K.T.); popov1rumen@gmail.com (R.P.); dr_eli@abv.bg (E.G.); 3 Department of Occupational Medicine, Faculty of Public Health, Medical University, 1527 Sofia, Bulgaria; tgk_70@abv.bg; 4 Department of Infectious Pathology, Hygiene, Technology and Control of Foods from Animal Origin, Faculty of Veterinary Medicine, University of Forestry, 1797 Sofia, Bulgaria; rpepovich@abv.bg; 5 Department of Microbiology, Infectious and Parasitic Diseases, Faculty of Veterinary Medicine, Trakia University, 6000 Stara Zagora, Bulgaria; ilia_tsachev@abv.bg

**Keywords:** hepatitis E virus, blood donors, seroprevalence, Bulgaria

## Abstract

Hepatitis E virus (HEV) infection is widespread among domestic pigs, industrial swine, and wild boars in Bulgaria. The aim of the current research was to present the HEV seroprevalence among blood donors in Bulgaria. In the present study, 555 blood donors (479 males and 76 females) were enrolled from five districts in the country (Shumen, Pleven, Stara Zagora, Plovdiv, and Sofia districts). All blood samples were tested for anti-HEV IgG using the *recom*Well HEV IgG ELISA test (Mikrogen GmbH, Neuried, Germany). Each participating donor completed a short, structured, and specific questionnaire to document data on the current study. Anti-HEV IgG positive results were detected in 144 (25.9%) blood donors, including 129 (26.9%) males and 15 (19.7%) females. The established HEV seropositivity was 28.8% (23/80) in Shumen district, 23.2% (22/95) in Pleven district, 27.1% (38/140) in Stara Zagora district, 27.5% (44/160) in Plovdiv district, and 21.3% (17/80) in Sofia district. A high HEV seroprevalence was found for persons who declared that they were general hunters (48.7%; 19/39; *p* = 0.001) and hunters of wild boars (51.6%; 16/31; *p* = 0.001). We present the first seroprevalence rates of HEV infection in blood donors from Bulgaria. The results of our research showed high HEV seropositivity among blood donors.

## 1. Introduction

The *Hepeviridae* family includes enterically-transmitted, small, non-enveloped positive-sense RNA viruses [1,2,3,4]. Members of this family are assigned to two genera: *Piscihepevirus* and *Orthohepevirus* [1,4]. The *Piscihepevirus* genus includes a single species whose typical isolate, prototype strain is cutthroat trout virus, which infects trout *Oncorhynchus clarkii* [5], although its pathogenicity and full host range are unknown [1]. The genus *Orthohepevirus* contains several viruses that infect a wide range of organisms, for example, humans, domestic pigs, wild boars, deer, sheep, rabbits, camels, and mongooses (*Orthohepevirus A* members); birds (*Orthohepevirus B* members); rats, ferrets, shrews, bandicoots, and mink (*Orthohepevirus C* members); and bats (*Orthohepevirus D* members) [1,6,7,8]. The *Orthohepevirus A* genus is divided into several genotypes: Hepatitis E virus (HEV) genotype 1 (gt1) and HEV gt2 have anthroponotic origins; HEV gt3 and HEV gt4 have zoonotic origins; HEV gt5 is probably zoonotic (infects wild boars and *Macaca fascicularis*); HEV gt6 infects wild boars; HEV gt7 is zoonotic (infects *Camelus dromedaries*, *Camelus bactrianus*, and humans); and HEV gt8 infects camels [6,7,8]. HEV gt1 is predominantly spread throughout Asia, the Middle East, and Africa; HEV gt2—Mexico and Africa; HEV gt3—Europe, North America, South America, and Asia; HEV gt4—Asia and Europe; HEV gt5 and HEV gt6—Japan; and HEV gt7 and HEV gt8—Asia and Africa [1,6,7,8]. In addition, unclassified viruses have been detected in moose and foxes, and in droppings from little egrets and kestrels [7]. Human HEV can cause acute virus infection in humans. HEV is mainly transmitted by contaminated water, as well as the consumption of undercooked or raw meat and other products from infected animals (most often from domestic pigs or wild boars) [9].

According to the World Health Organization (WHO) database, every year, there are approximately 20 million human HEV infections worldwide, leading to approximately 3.3 million symptomatic cases of HEV [10]. In this regard, WHO estimates that HEV caused 44,000 cases of lethal outcome in 2015 (accounting for 3.3% of the mortality due to viral hepatitis) [10].

The first HEV human cases were described in Bulgaria in 1995 [11]. Then, Teoharov et al. reported acute HEV infection in four cases in 53 tested patients [11]. For a period of about 20 years, there was a lack of research on HEV infection in humans and animals in the country. Since 2014, different studies on HEV infection in animals and humans have begun. Several studies have focused on hospitalized patients [12,13,14], outpatients [15], the general population [16,17], and hemodialysis patients [18]. Bulgarian researchers, in collaboration with Italian scientists, performed phylogenetic analysis of HEV isolated from people from Bulgaria [19,20]. In the last few years, the spread of HEV infection in animals in Bulgaria has been studied. Several seroprevalence studies on industrial pigs, domestic pigs, and wild boars have shown a high prevalence of HEV infection among animals in Bulgaria [21,22,23,24,25]. The established HEV seropositivity among industrial pigs varies from 40.0% to 60.3% [21,22,23], in domestic pigs—60.05% [24], and among wild boars—from 12.5% to 40.8% [24,25]. The prevalence of HEV in the only aboriginal pig breed (East Balkan swine) in Bulgaria was evaluated, and a high HEV seropositivity of 82.5% was found [26].

There is no available information on the HEV seroprevalence in Bulgarian blood donors. In this regard, we aimed to assess the current HEV seroprevalence rate among blood donors in Bulgaria. The present research gives new insights intothe epidemiology of HEV in one country from Southeastern Europe.

## 2. Materials and Methods

The current study was performed in accordance with the ethical principles of the Declaration of Helsinki (June 1964, last revision in October 2013). Blood samples were processed in accordance with the requirements of the Ministry of Health, Sofia, Bulgaria and complied with the good medical practices of the medical specialty “Transfusion Hematology” (part of the medical specialties in the Republic of Bulgaria). Informed consent was obtained from all blood donors included in the research. Information and approval documents were printed and signed by the blood donor and medical staff (physician or nurse). These documents were given to the medical staff and such documents are kept for 1 year. Medical staff checked that all the required documentation was complete and consent forms were signed before testing. Samples were discarded if the documents were incomplete or unsigned.

**Figure 1:**
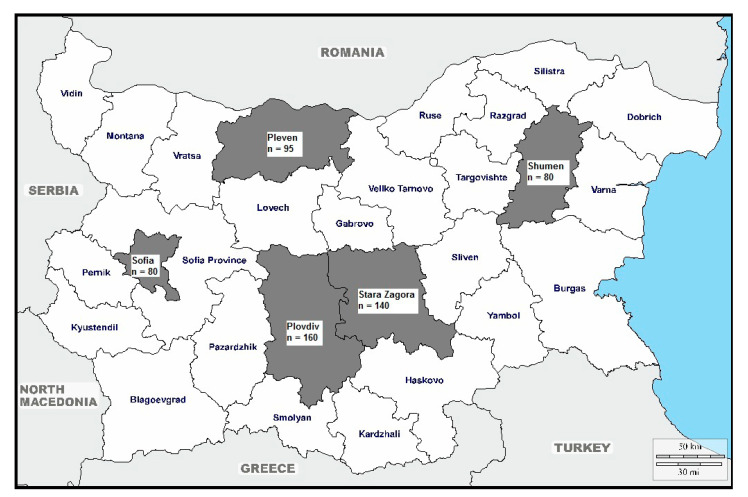
Geographic distribution of the Bulgarian blood donors by district (in gray).

The Bulgarian territory is divided into 28 administrative districts. Five hundred and fifty-five blood samples were collected from non-remunerated blood donor voluntaries (*n* = 555) enrolled from five districts (Figure 1). The geographic distribution of the investigated blood donors was spread across several large districts in Bulgaria, including Shumen (approximately 26°67′ E and 27°22′ E longitude; 42°99′ N and 43°61′ N latitude), Pleven (approx. 24°06′ E and 25°12′ E longitude; 43°16′ N and 43°64′ N latitude), Stara Zagora (approx. 25°11′ E and 25°87′ E longitude; 42°14′ N and 42°40′ N latitude), Plovdiv (approx. 24°48′ E and 25°12′ E longitude; 41°49′ N and 42°37′ N latitude), and Sofia (approx. 22°40′ E and 23°34′ E longitude; 42°34′ N and 42°80′ N latitude). The total population in these districts is as follows: Shumen, 172,262 people; Pleven, 236,305; Stara Zagora, 313,396; Plovdiv, 666,801; and Sofia, 1,328,790 [27]. The blood samples were collected from 1 June 2020 to 31 October 2020. The collection of blood samples was carried out through blood donation campaigns in the five districts of Bulgaria mentioned above.

Each participating donor completed a short, structured, and specific questionnaire to document data on the current study. The questionnaire contained information about demographics and baseline characteristics (sex, age, level of education, and area of residence), contact with animals (raising a domestic pig), the consumption of meat (pork meat, and meat from wild animals), derived meat products (pork sausage, salami, etc.), the consumption of seafood, hunting in nature (general hunting and hunting of wild boars). The questionnaire was validated by the Infectious Disease specialist and Transfusion Hematology specialist.

All samples were tested for anti-HEV IgG using *recom*Well HEV IgG ELISA kits (Mikrogen GmbH, Neuried, Germany). The *recom*Well HEV IgG is a qualitative and/or quantitative in vitro test employed for the detection and reliable identification of IgG antibodies to HEV in human serum or plasma. The *recom*Well HEV IgG is a screening test based on the principle of an indirect sandwich ELISA. In qualitative evaluation, the cutoff (limit) is the arithmetic mean that is calculated from the extinction values of both cutoff controls (at the beginning and end of the series). In quantitative evaluation, the corresponding antibody activity in units per mL is assigned to the extinction values using a formula. The measurement units U/mL are arbitrary units, which do not allow conclusions concerning (international) reference values. The *recom*Well HEV IgG ELISA test has a 98.9% sensitivity and 98.5% specificity.

All the data derived from the questionnaire were compared by the Chi-square test and *T*-test. The results on the seroprevalence of HEV infection in blood donors by district, part of the country, and region were compared by Fisher’s exact test and the Chi-square test. Binary logistic regression was used to evaluate the risk of positive results according to the part of the country and region. Statistical analysis was performed by MS Excel 2007 (Washington, DC, USA) and IBM SPSS Statistics version 21.0 (New York, NY, USA). A *p*-value < 0.05 was considered statistically significant.

## 3. Results

The overall mean age, mean male age, and mean female age were 37.2 ± 8.0 years (95% CI: 35.5–38.9), 37.1 ± 7.9 years (95% CI: 35.3–38.9), and 37.8 ± 8.8 years (95% CI: 32.6–43.0), respectively. The male sex dominated among analyzed blood donors (sex ratio: male/female = 1/0.13). People who had a low/intermediate level of education were 22.8% more frequently reported than participants who had high level of education (Table 1). In the group with a high level of education, men represented 78.0% (167/214) and women represented 22.0% (47/214) of the group, whilst in the group with a low/intermediate level of education, men represented 91.4% (312/341) and women represented 8.6% (29/341) of the group. The number of blood donors from a city was nearly nine times higher than that of blood donors from a village.

In total, 144/555 (25.9%) anti-HEV IgG positive samples were detected. The highest HEV seropositivity among the male sex was found in the age group ≥50 years (50.0%; 9/18). The highest anti-HEV IgG positive results among the female sex were found in the age groups of 30–39 years (27.5%; 8/29) and ≥50 years (25.0%; 2/8). No significant difference was found between HEV positive people with a high level of education (26.1%; 56/214; *p* > 0.05) and people with a low/intermediate level of education (25.8%; 88/341; *p* > 0.05). Similar levels of HEV seroprevalence were observed in participants from a city (25.8%; 129/500) and those from a village (27.2%; 15/55).

The highest levels of anti-HEV IgG positive results were found in Shumen district, and the lowest were recorded for Sofia county (Table 2). The distribution of HEV positive data in different age groups in the studied districts varied in different ranges. The mean HEV seropositivity in those aged 18–29 years old was 18.2% (95% CI: 11.0–25.4), the highest was found in Shumen county (32.0%), and the lowest was recorded in Sofia district (6.2%). The highest HEV seroprevalence in the age group 30–39 years was estimated in Pleven district (27.7%), the lowest was recorded in Sofia county (14.8%), and the overall percentage for all districts was 23.4% (95% CI: 19.5–27.3). The mean HEV seropositivity in those aged 40–49 years old was 27.2% (95% CI: 22.5–31.9), the highest was in found in Plovdiv county (35.8%), and the lowest was recorded in Sofia district (20.6%). The highest HEV positive results in older people (≥50 years) were calculated in Sofia county (54.4%), the lowest were recorded in Pleven district (0.0%), and the overall mean for all districts was 32.2% (95% CI: 16.6–47.8).

To estimate the risk for HEV seropositivity, the odds ratio (OR) in different parts of the country and regions was performed by binary logistic regression. The OR of anti-HEV antibody occurrence in Southern Bulgaria was determined in comparison to that in Northern Bulgaria (Table 3). We found that the odds of HEV infection were 1.018 times higher in Southern Bulgaria than in Northern Bulgaria. We estimated that the odds of HEV infection were 1.117 times higher in the North-Central region, 1.394 times higher in the South-Central region, and 1.495 times higher in the Northeastern region than in the Southwestern region.

The review of the questionnaire showed different results (Table 4). We did not find significant interactions between anti-HEV IgG positive results and the consumption of pork meat (*p* = 0.213); pork sausage, salami, etc. (*p* = 0.303); and seafood (*p* = 0.598). The breeding of domestic pigs was not found to be a significant factor for HEV seropositive among blood donors. A significant interaction was estimated between people who declared the consumption of meat from wild animals and anti-HEV IgG antibodies (37.8%; 45/119; *p* = 0.001). The statistical analysis showed significant results between general hunting and HEV seropositivity, as 48.7% (19/39; *p* = 0.001) of the general hunters had anti-HEV IgG positive results. Among hunters who declared that they go wild boar hunting, 51.6% (16/31; *p* = 0.001) of them were anti-HEV IgG positive participants.

## 4. Discussion

The HEV prevalence varies in different regions of the world. Mild and moderate HEV seropositivity among blood donors was observed in countries from the Balkan Peninsula. In Greece, an HEV positivity of 0.23% (6/2636) [28], 2.9% (36/1200) [29], and 9.43% (25/265) [30] was observed. Aydin et al. reported that out of 327 blood donors examined, 3 (0.92%) were anti-HEV IgG positive for HEV in Hacettepe University Medical Faculty Hospital (HUMFH), Turkey [31]. Petrovic et al. found an overall prevalence of 15.0% (30/200) for anti-HEV antibodies among blood donors from Novi Sad, Serbia [32]. In Croatia, the HEV seropositivity of 9.6% (99/1036; 95% CI: 7.9–11.5; ELISA test Mikrogen *recom*Well HEV IgG, old version) [33], 18.1% (188/1036; 95% CI: 15.9–20.6; ELISA test Mikrogen *recom*Well HEV IgG, new version) [33], 20.2% (209/1036; 95% CI: 17.8–22.7; ELISA test Dia.Pro HEV IgG) [33], and 20.3% (210/1036; ELISA test Dia.Pro HEV Ab) [34] was reported.

In other countries of the Mediterranean Basin, the prevalence of HEV infection was similar to that of the Balkan Peninsula (excluding France, where a much higher seropositivity was established). In Italy, a positivity of 8.7% (869/10,011; 95% CI: 8.14–9.25) [35], 9.1% (12/132) [36], and 9.8%/10.2%/17.4% (in various ELISA tests in one research) [37] was observed. In French blood donors, high HEV seropositivity values of 22.4% (2371/10,569; 95% CI: 21.6–23.2) [38], 54.4% (254/467) [39], and 56.1% (1518/2705; 95% CI: 54.2–58.0) [40] were found. In Iberian blood donors, mild and moderate anti-HEV IgG positive results of 2.5% (37/1473) [41], 2.8% (24/863) [42], 4.0% (2/50) [43], and 10.72%/19.96% (in two different ELISA tests in one study) [44] were observed.

In countries from Western Europe, the blood donor HEV seroprevalence varied widely. In Scotland, the reported seropositivity was 4.7% (73/1559; 95% CI: 3.7–5.8) [45]; Switzerland—4.9% (27/550) [46] and 20.4% (737/3609; 95% CI: 19.1–21.8) [47]; Ireland—5.3% (57/1076; 95% CI: 4.0–6.8) [48]; Germany—6.8% (69/1019; 95% CI: 5.3–8.3) [49]; Austria—13.55% (163/1203; 95% CI: 11.6–15.5) [50]; Norway—14.0% (162/1200) [51]; England—16.0% (80/500) [52]; and the Netherlands—31.0% (648/2100) [53]. In one Eastern European country (Poland), high anti-HEV IgG positive results of 43.52% (1340/3079; 95% CI: 41.78–45.28) [54] and 49.6% (122/246) [55] were observed.

The anti-HEV IgG positive result in the current research (25.9%) is similar to the results found in China (23.3% and 27.42%) [56,57], France (22.4%) [38], and Thailand (29.7%) [58]. The data from our study showed a high HEV seropositivity among blood donors from Bulgaria. We think that this could be influenced by various factors. One of the possible reasons for the result is the high HEV seropositivity among animals in the country. A high HEV prevalence has been observed among domestic pigs (60.05%) [24], industrial swine (40.0% and 60.3%) [21,23], wild boars (40.8%) [25], and East Balkan swine (aboriginal pig breed) (82.5%) [26] in Bulgaria. Consequently, meat and subproducts (sausage, salami, etc.) from these animals could be HEV-contaminated. The subsequent probable food-borne pathway may infect humans. This is one of the potentially possible explanations for our result. In this regard, we observed that a significant risk factor for HEV infection was the consumption of game meat (*p* = 0.001). Other authors have also reported that the consumption of raw and undercooked meat and meat products was a risk factor for HEV infection. Mansuy et al. found that HEV infection was more frequently observed in individuals eating pork meat, raw pork liver sausages (incl. figatelli), game meat, offal, and oysters [38]. Capai et al. reported that the highest risk for infection was the consumption of figatellu, pork meat, fittonu, sausages and pates, offal, liver, and wild berries [39]. Mooij et al. established that there was a direct connection between anti-HEV IgG seropositivity and eating traditional Dutch dry raw sausages (cervelaat, fijnkost, salametti, and salami), other dry sausages, smoked beef, steak, and raspberries among Dutch blood donors [53]. Among Nepalese blood donors, Shrestha et al. noted that pork consumption increased the risk for HEV infection [59].

Another possible reason for the high HEV seroprevalence among blood donors in Bulgaria (25.9%) could be the applied diagnostic ELISA test. Theoretically, it is possible, but we think that, in our study, this was unlikely, because the applied *recom*Well HEV IgG Mikrogen test a had high sensitivity (98.9%) and specificity (98.5%).

Regarding the HEV gender prevalence, we did not find a statistically significant sex difference in our study (*p* = 0.184). However, a review of the scientific literature showed that the HEV seropositivity was higher in males than in females [35,38,60,61,62].

In the present research, we observed that the highest HEV seropositivity was estimated among adults (40–49 years) and the lowest was found in younger participants (18–29 years). The results of the current research correspond to data presented by other authors. Spada et al. reported a 12% (552/4603; 95% CI: 11.09–12.96) anti-HEV IgG positive result in Italian blood donors older than 44 years versus 5.8% (254/4370; 95% CI: 5.16–6.55) for people younger than 44 years [35]. Mansuy et al. showed that the HEV IgG seroprevalence was higher in participants older than 45 years than in younger donors (30.7% vs. 14.7%; *p* < 0.001) [38]. Fearon et al. observed 9.9% HEV antibody positive donors among the age group 50+ years, and 1.6% among donors <29 years [60]. Takeda et al. noted a 6.4% (134/2100) positivity rate of IgG anti-HEV among Japanese blood donors in the age group 60–69 years versus 1.0% (21/2100) in the age group 20–29 years [61]. Shrestha et al. reported that the HEV IgG seropositivity was higher among participants ≥65 years (14.51%; 37/255; 95% CI: 10.19–18.83) than in the 25–34-year-old blood donors (2.28%; 13/569; 95% CI: 1.06–3.51) [62].

In the current study, we obtained the first preliminary results for HEV seroprevalence among hunters in Bulgaria. We found 48.7% (19/39; *p* = 0.001) anti-HEV IgG positive results among general hunters, and 51.6% (16/31; *p* = 0.001) among hunters of wild boars. In this regard, it is well-known that hunting is considered to be a risk factor for HEV infection. Lower levels of HEV seropositivity in hunters were observed by other authors. Among Estonian hunters, Ivanova et al. reported an analysis of sera from 144 hunters, which revealed the presence of HEV-specific IgG in 4.2% of the samples [63]. Schielke et al. analyzed 126 German hunters and 21% were positive for anti-HEV IgG antibodies (95% CI: 13.0–28.0) [64]. Baumann-Popczyk et al. showed that anti-HEV IgG results were identified in 22.2% (227/1021) of the studied Polish hunters [65]. Montagnaro et al. published an estimated HEV seroprevalence among Italian hunters (Latium region, Central Italy) of 25.0% (5/20; 95% CI: 6.1–43.9) [66].

The present study has some limitations that need to be addressed. Only the ELISA test was used for the detection of HEV IgG antibodies, without a confirmation test, and a confirmatory immunoblot test was not performed (including HEV RNA testing). In this regard, the results and conclusions should be interpreted with caution. Furthermore, the current research included several counties out of a total of 28 districts in the country, so it was not a nationwide survey. Despite these limitations, this work represents the first research on the anti-HEV IgG prevalence among blood donors in Bulgaria, enriching the knowledge on this infection for one country from Southeastern Europe.

## 5. Conclusions

We have presented the first seroprevalence rates of HEV infection in blood donors from Bulgaria. The results of the current study and those from previous investigations among animals show a high seroprevalence of HEV in the country. We think that this is probably linked to the autochthonous spread of HEV in the country, associated with the zoonotic characteristic of the virus. This hypothesis must be verified by future research on the molecular analysis of HEV in animals, humans, and food. It would be helpful to develop an information campaign to enrich people’s knowledge on HEV infection.

## Figures and Tables

**Table 1:** Seroprevalence of hepatitis E virus (HEV) infection by sex, age, education, and residence in blood donors from Bulgaria.

Characteristics	All Blood Donors (*n* = 555)	HEV Positive (*n* = 144)	HEV Negative (*n* = 411)	Statistics	df	*p*-Value
**Sex, *n* (%)**						
Female	76 (13.7)	15 (10.4)	61 (14.8)	1.77 ^X^	1	0.184
Male	479 (86.3)	129 (89.6)	350 (85.2)			
**Age, Years, Mean ± SD**	37.2 ± 8.0	38.1 ± 8.1	36.9 ± 7.9	1.63 ^T^	553	0.103
**Age Groups, *n* (%)**						
18–29	102 (18.4)	20 (13.9)	82 (20.0)	6.97 ^X^	3	0.073
30–39	209 (37.7)	51 (35.4)	158 (38.4)			
40–49	218 (39.3)	62 (43.1)	156 (38.0)			
≥50	26 (4.6)	11 (7.6)	15 (3.6)			
**Level of Education, *n* (%)**						
Low/Intermediate	341 (61.4)	88 (61.1)	253 (61.6)	0.000 ^X^	1	0.996
High	214 (38.6)	56 (38.9)	158 (38.4)			
**Area of Residence, *n* (%)**						
City	500 (90.1)	129 (89.6)	371 (90.3)	0.006 ^X^	1	0.941
Village	55 (9.9)	15 (10.4)	40 (9.7)			

**Note**: HEV = hepatitis E virus; df = degrees of freedom; and SD = standard deviation. Statistics: ^X^ = Chi-square test and ^T^ = *T*-test.

**Table 2:** Seroprevalence of HEV infection by district in blood donors from Bulgaria.

Districts	Investigated Blood Donors, *n*	HEV Positive, *n* (%)	Chi-Square	df	*p*-Value
**Northern Bulgaria**					
Shumen	80	23 (28.8)	0.85	1	0.356
Pleven	95	22 (23.2)			
**Southern Bulgaria**					
Stara Zagora	140	38 (27.1)	1.54	2	0.463
Plovdiv	160	44 (27.5)			
Sofia	80	17 (21.3)			

**Table 3:** Logistic regression showing the relationship between HEV positive blood donors and the geographic distribution.

Part of the Country/Region	Investigated Blood Donors, *n*	HEV Positive, *n* (%)	PE	SE	*p*-Value	OR	95% CI
**Part of the Country**							
Northern Bulgaria	175	45 (25.7)	NA	NA	NA	1.000	NA
Southern Bulgaria	380	99 (26.1)	0.018	0.209	0.933	1.018	0.676–1.532
Regions *							
Southwestern	80	17 (21.3)	NA	NA	NA	1.000	NA
North-Central	95	22 (23.2)	0.111	0.366	0.763	1.117	0.545–2.288
South-Central	300	82 (27.3)	0.332	0.302	0.272	1.394	0.771–2.522
Northeastern	80	23 (28.8)	0.402	0.368	0.275	1.495	0.726–3.078

Note: PE = parameter estimate; SE = standard error; OR = odds ratio; CI = confidence interval; and NA = not applicable. * Regions: Southwestern region = Sofia district; North-Central region = Pleven district; South-Central region = Stara Zagora and Plovdiv districts; and Northeastern region = Shumen district.

**Table 4:** Questionnaire analysis of HEV infection in blood donors from Bulgaria.

Answers	All Blood Donors (*n* = 555)	HEV Positive (*n* = 144)	HEV Negative (*n* = 411)	Chi-Square	df	*p*-Value
**Consumption of Pork Meat, *n* (%)**						
Yes	539 (97.1)	142 (98.6)	397 (96.6)	1.55	1	0.213
No	16 (2.9)	2 (1.4)	14 (3.4)			
**Consumption of Meat from Wild Animals, *n* (%)**						
Yes	119 (21.4)	45 (31.3)	74 (18.0)	11.10	1	0.001
No	436 (78.6)	99 (68.7)	337 (82.0)			
**Consumption of Pork Sausage, Salami, etc., *n* (%)**						
Yes	491 (88.5)	124 (86.1)	367 (89.3)	1.06	1	0.303
No	64 (11.5)	20 (13.9)	44 (10.7)			
**Consumption of Seafood, *n* (%)**						
Yes	294 (53.0)	79 (54.9)	215 (52.3)	0.28	1	0.598
No	261 (47.0)	65 (45.1)	196 (47.7)			
**Raising a Domestic Pig, *n* (%)**						
Yes	26 (4.7)	6 (4.2)	20 (4.9)	0.12	1	0.732
No	529 (95.3)	138 (95.8)	391 (95.1)			
**General Hunter, *n* (%)**						
Yes	39 (7.0)	19 (13.2)	20 (4.9)	11.32	1	0.001
No	516 (93.0)	125 (86.8)	391 (95.1)			
**Hunter of Wild Boars, *n* (%)**						
Yes	31 (5.6)	16 (11.1)	15 (3.7)	11.26	1	0.001
No	524 (94.4)	128 (88.9)	396 (96.3)			

## Data Availability

Not applicable.
